# Therapeutic Potential of Clove Oil in Mitigating Cadmium-Induced Hepatorenal Toxicity Through Antioxidant, Anti-Inflammatory, and Antiapoptotic Mechanisms

**DOI:** 10.3390/ph18010094

**Published:** 2025-01-14

**Authors:** Inas M. Elgharib, Fatma M. Abdelhamid, Gehad E. Elshopakey, Hatem Sembawa, Talat A. Albukhari, Waheed A. Filimban, Rehab M. Bagadood, Mohamed E. El-Boshy, Engy F. Risha

**Affiliations:** 1 Department of Clinical Pathology, Faculty of Veterinary Medicine, Mansoura University, Mansoura 35516, Egypt; menhm2010@gmail.com (I.M.E.); may_mokh@mans.edu.eg (F.M.A.); gehadelshopakey@mans.edu.eg (G.E.E.); elboshi@mans.edu.eg (M.E.E.-B.); 2 Department of Veterinary Diseases, Faculty of Veterinary Medicine, Delta University for Science and Technology, Gamasa 35712, Egypt; 3 Department of Surgery, Faculty of Medicine, Umm Alqura University, Makkah P.O. Box 7607, Saudi Arabia; hasembawa@uqu.edu.sa; 4 Department of Hematology and Immunology, Faculty of Medicine, Umm Alqura University, Makkah P.O. Box 7607, Saudi Arabia; tabukhari@uqu.edu.sa; 5 Pathology Department, Faculty of Medicine, Umm Al-Qura University, Makkah P.O. Box 7607, Saudi Arabia; wahfilim@gmail.com; 6 Department of Clinical Laboratory Sciences, Faculty of Applied Medical Sciences, Umm Al-Qura University, Makkah P.O. Box 7607, Saudi Arabia; rmbagadood@uqu.edu.sa

**Keywords:** clove oil, cadmium chloride, hepatorenal toxicity, oxidant/antioxidant status, apoptotic markers

## Abstract

Hazardous heavy metals, particularly cadmium (Cd), are widely distributed in the environment and cause oxidative stress in various animal and human organs. Clove oil (CLO), a common aromatic spice, has been used as a traditional medication as it has potent anti-inflammatory, antioxidant, and hepatoprotective properties. Background/Objectives: This study aimed to investigate the antioxidant, antiapoptotic, and anti-inflammatory effects of clove oil (CLO) against hepatorenal toxicity induced by cadmium (Cd). Methods: Twenty rats were equally divided into four groups: a control group, a Cd group treated with 15 mg/kg b.wt CdCl_2_, a CLO group administered 200 mg/kg b.wt CLO, and a Cd+CLO group. All groups were orally treated for 4 weeks. Results: Cadmium (Cd) exposure caused anemia and hepatorenal damage, as evidenced by increased serum levels of urea, creatinine, uric acid, total bilirubin (including its direct and indirect fractions), and elevated activities of liver enzymes such as alanine transaminase (ALT), aspartate transaminase (AST), and alkaline phosphatase (ALP). However, total protein and albumin levels decreased. Furthermore, there was a decrease in the levels of glutathione, glutathione transferase, and catalase in the liver antioxidant profiles. Meanwhile, malondialdehyde levels increased. Cadmium toxicity caused elevated expression of liver apoptosis markers, such as tumor necrosis factor-alpha (TNF-α) and caspase-3, and inflammation. CLO ameliorated the oxidative effects of Cd through decreasing urea (27.4%), creatinine (41.6%), liver enzymes, and hepatic apoptotic markers while increasing levels of total protein, albumin, and hepatic values of SOD (60.37%), CAT (64.49%), GSH (50.41%), and GST (9.16%). Conclusions: Hematological and biochemical parameters, as well as the antioxidant system, improved following clove oil treatment, leading to a reduction in hepatorenal damage. Therefore, it is possible to conclude that CLO protects rats from inflammation, apoptosis, and hepatorenal oxidative damage caused by Cd poisoning. Comprehensive translational research is required to validate CLO’s efficacy and safety of use in humans. Future studies should focus on elucidating the precise molecular mechanisms, optimal dosing strategies, and potential synergistic effects of CLO with other therapeutic agents.

## 1. Introduction

Cadmium (Cd) is a common heavy metal present in the environment that can be dangerous to humans and animals. It is involved in both natural and man-made processes, including geological weathering, the manufacture of batteries, pigments, and electroplating, the petrochemical, plastic, and refinery industries, and cigarette smoke. Cd mainly accumulates in the liver and kidney but also affects other organs [1]. It was classified in 1993 by the International Agency for Research on Cancer (IARC) as a Class I carcinogen [2]. The mechanism of Cd toxicity involves competition between Cd and essential metals, which modifies cellular processes, such as energy, metabolism, and metal membrane transport [3], or binding to thiol groups in the mitochondria, causing mitochondrial malfunction and damage [4]. After exposure, whether via ingestion or inhalation, Cd binds to blood cells, harming the hematological system and causing anemia [5]. The liver is the main organ for xenobiotic metabolism and detoxification. Reactive oxygen species (ROS) and the inhibition of hepatic antioxidant enzyme activity are produced when Cd is bound to hepatic cell membranes. This results in an increase in total bilirubin and liver functional enzymes (ALT, AST, and ALP) and a decrease in total protein levels and lipid and lipoprotein production in the liver, causing endoplasmic reticulum stress and increasing cell death by raising the levels of reactive oxygen radicals. Cd exposure promotes tissue damage caused by lipid peroxidation [6], and according to Andjelkovic et al. [7], Cd weakens the antioxidant enzyme system, causing oxidative stress due to altered gene expression. Cd is also an immunotoxin inhibitor that damages the immune system and causes apoptosis by directly interacting with immune cells, changing their status and function, and increasing within them [8]. Studies suggest that Cd may also harm the liver by elevating TNF-α production [9]. According to Abdelrazek et al. [10], as an essential component of apoptosis, caspase-3 is upregulated under hazardous conditions, such as Cd toxicity, and is the last step in many apoptotic pathways. However, the molecular mechanisms behind the toxic effects of cadmium in the liver and kidney are still not fully understood.

For millennia, people have utilized CLO, a common aromatic spice, as a traditional medication to treat fever, anemia, diarrhea, coughing, and dental discomfort [11] and as a food preservative. CLO possesses antioxidant properties, depending on the source. Additionally, it is among the greatest sources of phenolic chemicals, including gallic acid, eugenol acetate, and eugenol [12]. CLO is also a potent free radical scavenger that donates hydrogen atoms via its -OH groups [13]. Phytochemical investigations into CLO confirm its notable concentration of phenolic and flavonoid components and the positive relationship between its antioxidant capacity and phenolic and flavonoid levels. According to Soliman et al. [14], the amounts of these compounds vary depending on the type of plant material and the chemical composition of the compounds that can be extracted [15]. The extracts’ overall lowering power and capacity to scavenge DPPH, which are influenced by the extraction solvent, were employed to assess their level of antioxidant activity [16]. In the current investigation, the main components of CLO were recovered using GC-MC, which is considered the best analytical method for volatile oil [17]. We obtained 76% eugenol, 17.4% caryophyllene, 2.12% alpha humulene, and 1.2% eugenol acetate. However, according to another study by Bakour et al. [18], the main ingredients are eugenol and eugenol acetate. This variation could be attributed to a variety of factors, including meteorological conditions, soil composition, genetic characteristics, age, maturity stage, plant section type, and distillation techniques [19]. The amount of volatile metabolites in the oil can vary depending on the conditions of distillation and storage. The extraction technique utilized to produce the oil can also have an impact on its chemical makeup [20]. Nevertheless, CLO possesses potent anti-inflammatory, antioxidant, and hepatoprotective properties. Two important aspects of its anti-inflammatory action are the inhibition of TNF-α and the reduced production of nitric oxide (NO) radicals. [21]. Furthermore, its effectiveness against cancer has recently been confirmed [22]. The safe use of clove oil has also been confirmed by the American Food and Drug Administration (FDA), with 2.5 mg/kg body weight being the recommended daily consumption according to the World Health Organization (WHO) [23]. Ugenol is the primary phytochemical recovered from CLO with a potent antioxidant effect, with a total level of nearly 80%, depending on the extraction method. Its constituents are alpha-humulene, eugenol acetate, and beta-caryophyllene [24].

Our study is groundbreaking in its attempt to bridge a critical knowledge gap. Unlike previous research, which predominantly focused on the general antioxidant properties of clove oil, as well as its molecular modulation of cadmium-induced pathophysiology, this study lays the foundation for its potential therapeutic application against cadmium toxicity by elucidating how CLO interacts with key oxidative, apoptotic, and inflammatory pathways. Furthermore, CLO, as a natural, readily available, and cost-effective remedy, offers a promising alternative that warrants thorough investigation; thus, this study represents a critical step toward the development of novel interventions for combating cadmium-induced hepatorenal damage.

## 2. Results

### 2.1. Phytochemical Analysis and Antioxidant Activity of Clove Oil

The total phenolic content of clove oil was 172.38 mg gallic acid equivalent/100 g, as shown in Table 1. The standard flavonoid content for CLO was 54.358 mg catechin per 100 g. As presented in Table 2, the antioxidant capacity of CLO was calculated as the percentage of residual 2,2-diphenyl-1-picrylhydrazyl (DPPH), which ranged from 49.72 to 85.23, and the lowest percentage of remaining DPPH (49.72) was associated with the highest concentration of CLO (0.168 mg/mL).

### 2.2. CLO Analysis Using Gas Chromatography–Mass Spectroscopy (GC–MS)

Table 3 shows that 23 known elements of CLO were examined. There were four main substances, with eugenol having the highest concentration at 16 min retention time (RT) and beta-caryophyllene having the lowest concentration at 18 min RT. Alpha humulene presented a 2.1% concentration at 19 min RT, and eugenol acetate presented 1.2% at 21 min RT.

### 2.3. Hematological Result

When the Cd-intoxicated group was compared to the control group, the hematological results showed a significant decrease in packed cell volume (PCV%), hemoglobin (Hb g/dL), and red blood cell count (RBCs × 10^6^), while total leukocyte count was increased in the Cd group relative to the NC group. Simultaneously, the group treatment with Cd+CLO showed a marked improvement in its hematological profile compared to the Cd group (Table 4). Differences in the blood indices MCV, MCH, and MCHC were insignificant across all study groups (Table 4).

### 2.4. Liver and Kidney Function Biomarkers

**Figure 1:**
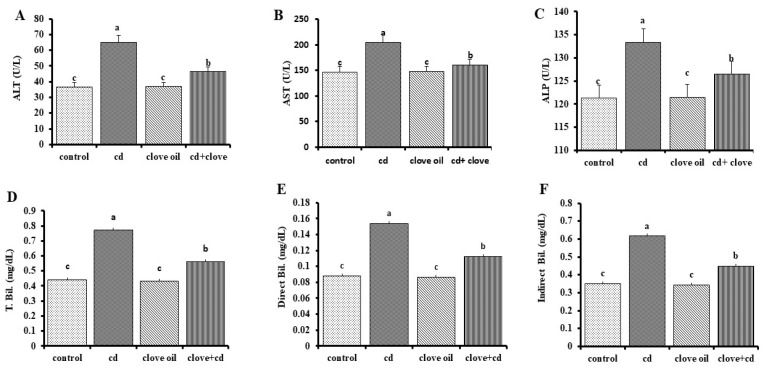
Liver function tests at fourth week after clove oil treatment in rats intoxicated with cadmium (mean ± SE). Significant values are defined as those with distinct superscript letters (*p* < 0.05). The enzymes that convert amino acids to bilirubin are (**A**) alanine aminotransferase, (**B**) aspartate aminotransferase, (**C**) alkaline phosphatase, (**D**) total bilirubin, (**E**) direct bilirubin, and (**F**) indirect bilirubin.

**Figure 2:**
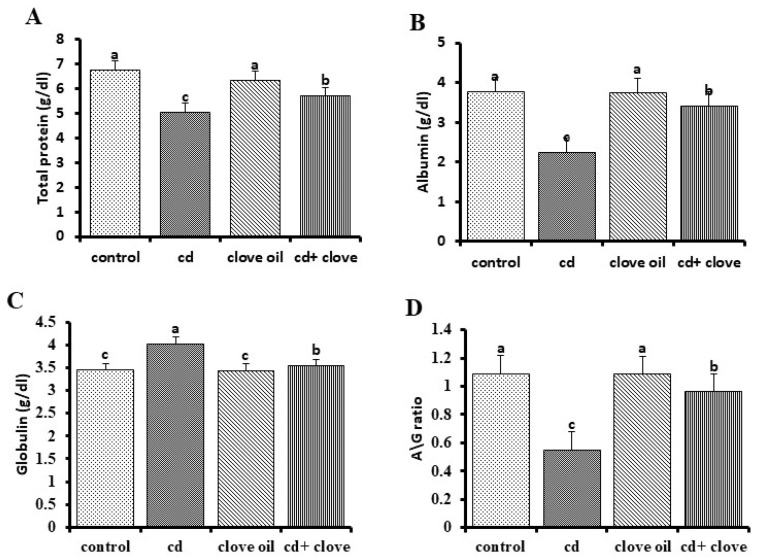
Proteinogram of rats intoxicated with cadmium at the end of the fourth week after receiving clove oil treatment (mean ± SE). Significant values are defined as those with distinct superscript letters (*p* < 0.05). (**A**) Globulin, (**B**) albumin, (**C**) total protein, and (**D**) A/G ratio.

The serum blood levels of ALT, AST, and ALP together with total (TB), direct, and indirect bilirubin with globulin were significantly higher, while the serum total protein and albumin were distinctly reduced in the Cd group compared to the NC group (Figure 1 and Figure 2, *p* < 0.05 for all). Both Cd+CLO treatment groups presented substantial reductions in the blood enzyme activities of ALT, AST, and ALP with total and direct bilirubin, simultaneously with increased blood total protein and albumin levels, compared to the Cd group (*p* < 0.05 for all markers). Furthermore, all tested liver function markers were equal between the CLO and control groups (Figure 1 and Figure 2). As shown in Table 5, rat kidneys were negatively impacted by cadmium toxicity, as shown by the markedly higher serum levels of urea, uric acid, and creatinine in Cd-intoxicated rats compared to the control group. However, treatment with CLO significantly reduced the renal damage caused by Cd and decreased urea, uric acid, and creatinine levels, although they did not return to normal.

### 2.5. Hepatic Antioxidant and Oxidative Markers

In contrast to the control group, Cd toxicity caused hepatic oxidative stress, as shown by a significant increase in hepatic MDA and a significant decrease in SOD, CAT, GSH, and GST antioxidant molecules (*p* < 0.05 for all) (Table 6). Compared to Cd+CLO intoxicated rats, the Cd group displayed a significant reduction in hepatic MDA with increased hepatic concentrations of SOD, CAT, GSH, and GST. In all tests, the antioxidant and oxidative markers were equal between the CLO and NC groups, except for GST activity, which was significantly higher in the CLO group (Table 6).

### 2.6. Cadmium Residues in Experimental Rat Liver

Table 7 provides a summary of the findings regarding Cd residues in the liver. Rats exposed to Cd had higher levels of Cd residues than control rats; however, when CLO was also administered, the concentration of Cd was significantly lower than that in the Cd-only group. There was no discernible difference in the levels of Cd residues in the liver between the rats treated with CLO and control rats.

### 2.7. Histopathological Investigation

#### 2.7.1. Liver

**Figure 3:**
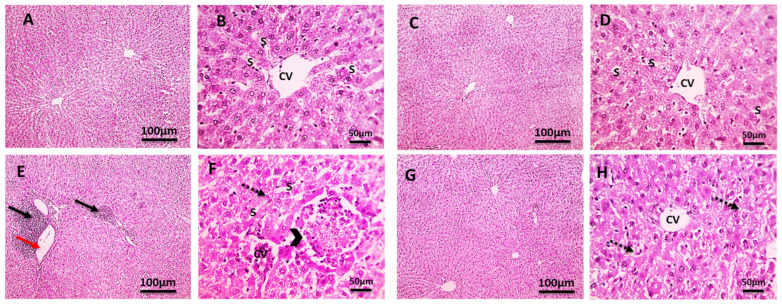
Microscopic images of H&E-stained hepatic sections from the control group (**A**,**B**) and CLO group (**C**,**D**) demonstrating typical radially oriented hepatic cords around the central veins (CV) with normal portal regions and sinusoids (s). H&E-stained hepatic sections from the Cd group (**E**,**F**) demonstrating focal areas of coagulative necrosis (black arrowheads), vascular dilation (red arrow), marked inflammation (black arrows) in the portal areas, and hydropic degeneration in hepatocytes (dashed black arrows). H&E-stained hepatic sections from the Cd+CLO group (**G**,**H**) demonstrating hydropic degeneration in hepatocytes (dashed black arrows) (100 bar 100 is the low magnification, and 400 bar 50 is the high magnification).

As shown in Figure 3, normal hepatic parenchyma was observed in the control group (Figure 3A,B) and CLO group (Figure 3C,D). Several normal hepatic lobules split by very thin layers of connective tissue septa were also observed. Normal hepatic cords protruded from the thin-walled core veins of each normal hepatic lobule. A typical portal region, which includes the bile ducts, hepatic artery, and portal vein, was also present. The hepatic sections of the Cd group (Figure 3E,F) showed intense portal inflammation, hydropic hepatocyte degeneration, isolated areas of coagulative necrosis with leukocytic cells, obstructed sinusoids, and central veins with leukocytic cells. These alterations decreased in the Cd+CLO group (Figure 3G,H). The hepatocyte patterns were normal; there was less inflammation in the portal region and few leukocytic cells surrounding the CV in the hepatic sections (Figure 3).

#### 2.7.2. Kidney

**Figure 4:**
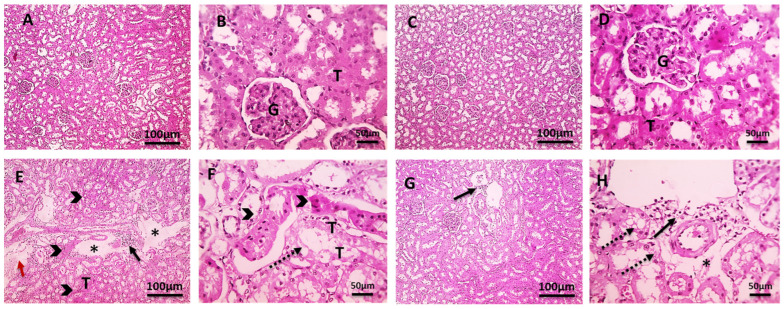
Microscopic images of H&E-stained renal cortical sections revealing normal tubules (T) and glomeruli (G) with minimal interstitial tissue in the control group (**A**,**B**) and CLO group (**C**,**D**). H&E-stained renal cortical sections from the Cd group (**E**,**F**) revealing severe hydropic degeneration of the tubular epithelium (dashed black arrows), necrosis (black arrowheads), congestion (red arrows), and marked perivascular edema (*) with many mononuclear cells infiltrating the interstitial tissue (thin black arrows). H&E-stained renal cortical sections from the Cd+CLO group (**G**,**H**) showing minor perivascular edema (*) and milder hydropic degeneration in a few tubules (dashed black arrows) and some mononuclear cells infiltrating the interstitial tissue (thin black arrows) (100 bar 100 is the low magnification, and 400 bar 50 is the high magnification).

As demonstrated in Figure 4 renal cortical slices from the control group (Figure 4A,B) and CLO group (Figure 4C,D) showed typical tubules and glomeruli with little interstitial tissue. The renal cortical sections of the Cd group (Figure 4E,F) exhibited significant hydropic degeneration of the tubular epithelium, necrosis, congestion, perivascular edema, bleeding, and the infiltration of mononuclear cells in the interstitial tissue. The histological appearance of the renal cortical sections in the Cd+CLO group (Figure 4G,H) showed some improvement, with decreased tubular epithelial hydropic degeneration. Mild perivascular edema and a few interstitial lymphocytic cell infiltrations were observed in a few sections.

### 2.8. Immunohistochemical Expression of Caspase-3 and TNF-α

**Figure 5:**
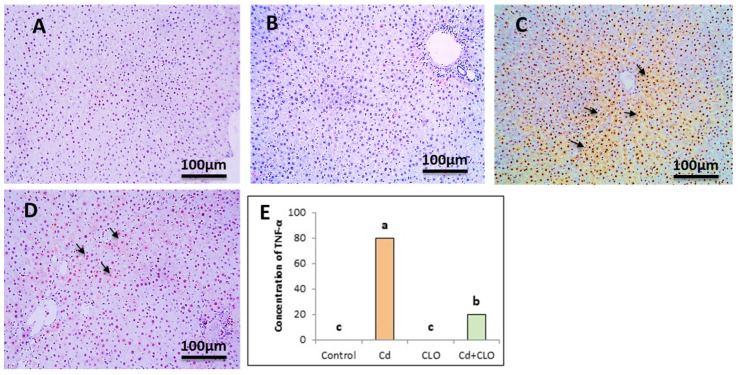
Immunostained liver section displaying negative immunostaining against caspase-3 in the control group (**A**) and CLO group (**B**). Liver displayed strong immunostaining against caspase-3 in the Cd group (**C**). Liver sections displayed a marked decrease in immunostaining against caspase-3 in the Cd+CLO group (**D**). (IHC, DAB immunostaining, hematoxylin as a counterstain, 100×). Bars (**E**) represent the scores of caspase-3 expressions (mean ± SE). Values with different superscript letters are considered significant at *p* < 0.05.

Liver tissue sections from all the rat groups were immunostained with caspase-3 and TNF-α using immunohistochemical labeling. The control and CLO groups exhibited negative immunostaining for both caspase-3 and TNF-α markers. When rats were exposed to Cd toxicity, the levels of caspase-3 displayed strong positive brown immunostaining against caspase-3 in the cytoplasm of hepatocytes. Liver tissue from the Cd+CLO group displayed a marked decrease in positive brown immunostaining against caspase-3 in the cytoplasm of hepatocytes. The statistical analysis showed a significant increase in caspase-3 in the Cd group compared to the control and CLO groups. A significant decrease was apparent in the Cd+CLO group compared to the others (Figure 5).

**Figure 6:**
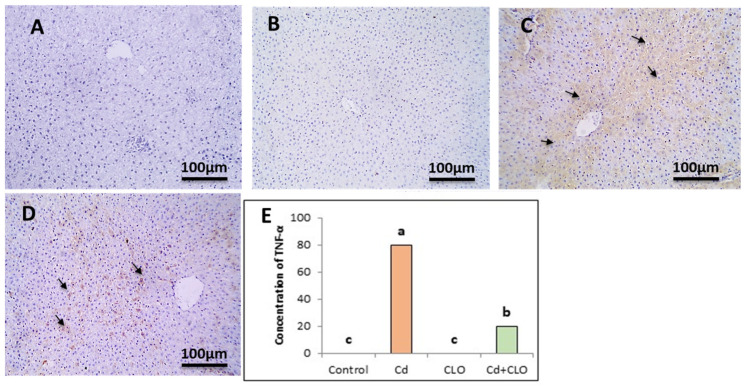
Immunostained liver section displaying negative immunostaining against TNF-α in the control group (**A**) and CLO group (**B**). Liver displayed strong immunostaining against TNF-α in the Cd group (**C**). Liver sections displayed a marked decrease in immunostaining against TNF-α in the Cd+CLO group (**D**) (IHC, DAB immunostaining, hematoxylin as a counterstain, 100×). Bars (**E**) represent the scores of TNF-α expression (mean ± SE). Values with different superscript letters are considered significant at *p* < 0.05.

When rats were exposed to Cd toxicity, the levels of TNF-α displayed a strong positive brown immunostaining against TNF-α in the cytoplasm of hepatocytes. Liver tissue from the Cd+CLO group displayed a marked decrease in positive brown immunostaining against TNF-α in the cytoplasm of hepatocytes. The statistical analysis showed a significant increase in TNF-α in the Cd group compared to the control and CLO groups. A significant decrease was apparent in the Cd+CLO group compared to the others (Figure 6).

## 3. Discussion

Cd is a heavy metal that affects the endoplasmic reticulum, cell membrane, mitochondria, lysosomes, and a number of enzymes involved in metabolism, detoxification, and damage repair. Cd-induced hepatotoxicity is a well-known reason for liver damage in both humans and animals, according to Rana et al. [25]. CLO (*Syzygium aromaticum*) is one of the most well-known essential oils to have antioxidant properties, depending on its source. Additionally, it is also among the greatest sources of phenolic compounds, including gallic acid, eugenol acetate, and eugenol [12]. It is a potent free-radical scavenger that donates a hydrogen atom via its -OH groups. The effects of Cd administration in the biological system depend on the route of exposure, the dose administered, and the duration of exposure. The maximum rate of Cd accumulation was found to be up to 70%, recorded in the cytoplasm, followed by 15% in the nucleus, with relatively low quantities recorded in the endoplasmic reticulum and mitochondria [26]. According to Soliman, Ibrahim, and El Baz [14], Cd is an accumulative toxin in various organs, especially in the liver of rats, as was found in our research, due to the presence of high concentrations of MT (metallothionine)—a finding in agreement with Nordberg and Nordberg [27]. MT in the liver possesses a high affinity for Cd ions [28]. A deficiency in MT results in hepatic injury and accumulation. Moreover, the amount of Cd that accumulates in the body varies on the exposure’s route, dose, and duration [29]. Cd treatment with CLO reduced the Cd accumulation in hepatic cells because they contain hydroxyl (-OH) and carbonyl (C=O) functional groups that compete with Cd for sulfhydryl-binding sites on metallothionine [30]. The considerable decline observed in this study could point to the detrimental effects of Cd on RBC count, Hb concentration, and PCV%. In addition, we found no significant changes in MCV, MCHC, and MCH, indicating that Cd may cause normocytic normochromic anemia even after acute exposure, thought to be caused by a defect in the synthesis of erythropoietin, as Cd directly causes bone marrow mesenchymal stem cell (BMSC) apoptosis [31]. Additionally, our results showed that Cd toxicity caused a significant increase in WBC count compared to modulators; this result in the control group, which may be indicative of early induction of inflammatory responses to external immunity, is consistent with the results of [32]. On the other hand, Cd toxicity induced a significant decrease in WBC count. The CLO co-treatment improved RBC count, Hb conc, and PCV%, along with a significant decline in WBC count, compared to the Cd-intoxicated group due to its anti-inflammatory effects in the lymphoid systems and kidney; this, in turn, enhanced the reproduction of the erythropoietin hormone, which controls the generation of red blood cells and their maturation in bone marrow [33].

Cd is a potent hepatotoxic agent [34]. In the current investigation, the changes in total protein and albumin contents in Cd-exposed rat livers showed a loss of the liver’s protein synthesis capabilities due to mitochondrial and cytosolic dysfunctions [35]. In contrast, serum globulin levels increased somewhat more than in the control group due to its oxidative action, which stimulated the immune system to produce more globulin as a defense mechanism [36]. The A/G ratio in Cd-intoxicated rats was much lower than that in the control groups, owing to globulin exceeding that of albumin, with the CLO treatment regenerating damaged cells in the liver [37]. The most recent data demonstrate a sharp rise in serum liver enzymes and ALP after the fourth week of Cd poisoning, indicating liver damage and the loss of the integrity of the functioning membrane, as well as hepatic enzyme leakage into the blood [38]. These variations in hepatic function are supported by histological changes in rat liver cells after Cd intoxication, including the loss of hepatic architecture and modifications to the lipid structures of liver tissue. Cell membrane rupture, cytoplasmic vacuolization, and nuclear pyknosis may be caused by the production of highly reactive radicals and subsequent lipid peroxidation. In the current study, co-treatment with CLO stabilized the cell membrane, which resulted in a significant drop in serum ALT, AST, and ALP levels [39]. Elevated serum bilirubin levels are a clear marker of liver damage [40]. Furthermore, our study revealed that increased serum total and direct bilirubin, which is a specific sign of liver damage, may be due to increased hepatocyte damage, inflammatory cell infiltration, and biliary tract dysfunction, which inhibit hepatocytes from absorbing bilirubin and the accumulation of fat droplets in Cd-treated rats [41]. The co-treatment of CLO with Cd in the present investigation decreased bilirubin levels by ameliorating the synthetic function and hepatic excretory mechanism. Cd is a classic nephrotoxin [42]. According to our study, a significant elevation in urea, creatinine, and uric acid was observed in the Cd-intoxicated group compared to the control group, which was confirmed by the severe histopathological changes in renal tissue, consistent with the findings of Gabr et al. [43] and Yang and Shu [44]. Proximal tubule cell interactions with low-molecular-weight thiols (cysteine and glutathione) may be the pathophysiology of the harmful effects on the kidney [45]. Moreover, altered DNA methylation patterns, the suppression of DNA repair processes, and disruption of Na^+^-K^+^-ATPase have all been implicated in Cd-induced nephrotoxicity [46]. Co-treatment with CLO led to decreased values in the kidney function tests compared to the Cd-treated group, which is in agreement with the results of Hussein et al. [47]. Flavonoids act as diuretics, increasing the glomerular filtration rate [48].

In the present study, exposure to Cd in the Cd-intoxicated group showed a significant decrease in the GSH level and CAT, SOD, and GST activity in hepatic homogenates compared to the control group, indicating the presence of oxidative stress, as clarified in a study by Wang et al. [49]. In addition, there was a significant elevation in hepatic MDA in rats compared to the control group, in agreement with Aja et al. [50]. The oxidative effect of Cd may be due to the removal of cations from the active sites of several antioxidant enzymes, such as SOD, CAT, and GPx [51], which compete with metals in these antioxidant enzymes. For example, when Cd replaces selenium in GPx or zinc in SOD, the antioxidant activity of these enzymes is reduced or lost [52]. Co-treatment with CLO caused a significant elevation of CAT, SOD, GST, and GSH, accompanied by a decline in MDA, compared to the Cd-intoxicated group, via the hydroxyl groups in the eugenol transfer electrons or hydrogen atoms to neutralize free radicals, blocking the oxidative process. In addition, Cd inhibits iron-mediated lipid peroxidation and the auto-oxidation of Fe^2+^ ions [53]. Rats treated with CLO alone showed a considerably higher GST activity than rats in the control group, indicating an improvement in the antioxidant defense system. It is well established that Cd induction can produce histopathological alterations and changes in lipid composition, causing macrophages to release TNF-α (a cytokine that promotes inflammation produced by Kupffer cells) as a defensive mechanism. However, when cells are subjected to severe infection or toxicity, NO is produced in high quantities and can cause a pathological state via NO-induced oxidative stress [54]. In the current study, the Cd-treated group showed a significant increase in TNF-α expression compared to the control group when immune histochemical techniques were applied in [55]. Enhanced oxidative stress can disrupt the mitochondrial membrane’s permeability, releasing cytochrome C into the cytoplasm, where it attaches to Apaf-1 (apoptotic protease activating factor 1) and initiates the cascade that ends in cell death [56]. In the present study, the treatment with CLO caused a significant reduction in TNF-α expression to near the control level due to the anti-inflammatory properties of CLO via the inhibition of the NF-kB factor (nuclear factor kappa light chain enhancer of activated B cells), a family of highly conserved transcription factors that modulate inflammatory responses, cellular proliferation, and death by activating TNF-α and decreasing the production of cyclooxygenase (COX)-2 in macrophage-stimulated lipopolysaccharide (LPS).

Apoptosis, or programmed cell death, is essential for multicellular organism survival because it eliminates damaged or infected cells, which may interfere with normal function [57]. It can be regulated by many modulators and proteins (caspases) [58] stimulated by Cd. Caspase-3 is a crucial molecule for regulating both mitochondrial and death receptor apoptotic pathways [59] and is more specific in the detection of apoptotic cells [60]. According to our study, caspase-3 activity was significantly enhanced in the liver of the Cd-intoxicated group compared to the control. This may be because Cd can increase intracellular ROS, resulting in cellular oxidative stress, and activate the mitogen-activated protein kinase (MAPK)-family-associated proteins (which control how cells react to a variety of stimuli, such as proinflammatory cytokines, mitogens, osmotic stress, and heat shock). They regulate a variety of cellular processes, including apoptosis, mitosis, differentiation, gene expression, and proliferation [61]. This is mediated by abnormal mitochondrial membrane potential and the expression of apoptosis-related proteins such as Bax, Bcl-2, and cleaved caspase-3. Along with the co-treatment with CLO causing a significant decline in caspase-3 compared to the group intoxicated with Cd, our result agreed with Abadi et al. [62]. This may be due to the scavenging of the generated ROS, with the potent antioxidant effect preventing the release of Ca^+2^ from the endoplasmic reticulum into the cytosol, as well as the release of cytochrome C, which ultimately prompts the activation of cell death. CLO also regulates the apoptotic and anti-apoptotic protein ratio (Bax/Bcl2 ratio), which causes a decrease in cleaved caspase-3 expression. Recent research has clarified the important role of clove oil in decreasing the dangerous effects of COVID-19, such as pneumonia and lung thrombosis, via the inhibition of thrombin-induced platelet aggregation and improving the blood supply of both the brain and heart [63]. Other research has proven that clove oil has cytotoxic activity against oral, prostate, lung, brain, and breast cancer due to anti-proliferative capabilities [64]. The most relevant contribution of our study is understanding the therapeutic potential of clove oil (CLO) in mitigating cadmium toxicity, addressing a critical knowledge gap in the current research. While previous studies have primarily focused on the general antioxidant properties of CLO and its molecular modulation of cadmium-induced pathophysiology, this research takes a more comprehensive approach. By investigating how CLO interacts with key biological pathways, including oxidative stress, apoptosis, and inflammation, we provide new insights into its potential to counteract the toxic effects of cadmium exposure. Unlike conventional treatments, CLO is a natural, readily available, and cost-effective remedy, making it a highly promising alternative for therapeutic use. Our findings lay the foundation for the further exploration of CLO as a novel intervention to combat cadmium-induced hepatorenal damage. This study represents a critical first step toward developing more accessible and effective strategies to prevent and treat cadmium toxicity, offering hope for a safer, sustainable approach to managing environmental toxin exposure.

Building upon the findings of this study, there are several critical areas for future investigation to further understand and harness the potential of CLO in mitigating cadmium toxicity via the study of the precise molecular mechanisms through which clove oil modulates oxidative stress and inflammation caused by cadmium exposure. In addition, exploring the role of CLO in chelation and excretion pathways to determine its efficacy in reducing cadmium accumulation in tissues is of importance. Moreover, conducting dose–response studies to identify the most effective and safest concentrations of clove oil for mitigating cadmium-induced toxicity across different biological systems is desirable. Also, further study on the evaluation of the long-term effects of clove oil on chronic cadmium exposure, especially in populations at risk of prolonged environmental or occupational exposure, is required, as is a comparative study on the efficacy of clove oil with other known natural antioxidants or pharmacological interventions in mitigating cadmium toxicity. Our study’s use of CLO as crude oil presents a limitation, and more research should be conducted using this oil’s active components. Another restriction is using a single dose of CLO, where multiple doses should be administered. Future studies should be applied to demonstrate that CLO has an effective hepatorenal protective effect by using purified active components and varied dosages of this oil. Furthermore, subsequent work is necessary to strengthen the understanding of the underlying mechanisms of how these protective effects occur. Nevertheless, further investigations are required to assess and compare the efficacy and safety of CLO in both prophylactic and therapeutic uses for hepatorenal damage related to chronic heavy metal toxicity.

## 4. Materials and Methods

### 4.1. Chemicals

Dimethyl sulfoxide, CLO, and cadmium chloride (Cdcl2) were acquired from Sigma Business, Egypt. The kits for SOD, MDA, GSH, catalase, and GST reagents were purchased from the Biodiagnostic Company, Egypt. The kits for determining total protein, AST, ALT, and ALP levels for each group of individuals were acquired from Diamond Company, Scy Tek Laboratories, and Diagnostic Biosystems for caspase-3 and TNF-α, respectively.

### 4.2. Phytochemical Composition of Clove Oil

#### 4.2.1. Total Amount of Phenolics

Gallic acid was used as a standard in the application of the Folin–Ciocalteu technique, which measures the total phenolic content. The total phenolic content of the oil sample was determined and reported as milligrams of gallic acid equivalent per gram of oil sample weight based on the standard curve (y = 0.0062x, r2 = 0.987) [65].

#### 4.2.2. Total Amount of Flavonoids

Using aluminum chloride as a reference standard for catechin, a colorimetric examination was performed to determine the total flavonoid content. The weight of the oil sample and standard curve (y = 0.0028x, r2 = 0.988) were used to calculate the amount [66].

#### 4.2.3. Assay for Antioxidant Activity 2,2-Diphenyl-1-Picrylhydrazyl (DPPH)

Ascorbic acid was used as a reference because we examined the antioxidant capacity of the oil sample using the DPPH• colorimetric method by way of the assay reported by Kitts et al. [67]. Briefly, an equivalent amount of each sample was combined with methanol to prepare a serial dilution. A DPPH solution (0.135 mM concentration) was prepared, and 1 mL was added to each sample prior to serial dilution. The mixture was left in the dark at room temperature for 30 min after the addition of the DPPH solution. Next, the absorbance of each sample was measured at a wavelength of 517 nm. The % of DPPH• remaining was calculated using the following equation: [DPPH•] [T/DPPH•]T = 0 × 100. The values were then plotted aversely to the milligrams of oil sample/mL using an exponential curve to identify the effective concentration (IC50). IC50 indicates the level of antioxidants needed to decrease the initial concentration of the DPPH• solution by 50%. The IC50 values indicated an inverse relationship with the antioxidant capacity of the tested samples.

#### 4.2.4. Gas Chromatography–Mass Spectroscopy (GC–MS) of Clove Oil Samples

We utilized an Agilent 5975C and QP2010-GC–MS with an autosampler equipped with a capillary column (automatic injector high-purity helium as the carrier gas; injector temperature maintained at 280 °C, split mode (1:10)), followed by an initial temperature of 40 °C and a final temperature of 300 °C and an initial time of 5 min and a final time of 7.5 min at 8/min [68].

### 4.3. Animals and Experimental Protocol

**Figure 7:**
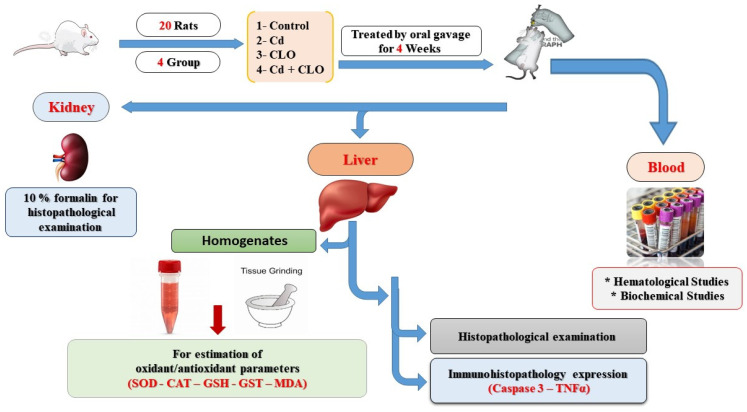
Experiment design.

Twenty-six-week-old male albino rats, weighing between 150 and 180 g, were acquired from Tanta Experimental Animal House and housed in 50 × 30 × 25 cm plastic boxes with mesh wire covers for a period of 15 days to allow for adjustment. The recorded room temperature was 25 ± 2 °C, with a 12 h light/dark cycle and 60–70% humidity. Rats were provided with a pellet diet and hydrated with fresh water. The Animal Research Ethical Committee of the Mansoura Faculty of Veterinary Medicine was involved in this study. Following a two-week acclimatization period, twenty rats were divided into four equal groups of five rats each, as depicted in Figure 1. Control group rats were administered normal saline by oral gavage. Cd group rats were administered an oral gavage dose of 15 mg/kg of Cdcl_2_ [69]. CLO group rats were given clove oil (CLO) dissolved in DEMSO at a dose of 200 mg/kg b.wt orally via gavage, as per [70]. The Cd+CLO group was given identical dosages of Cdcl_2_ and clove oil every day. All treatments were continued for four weeks (Figure 7).

### 4.4. Sampling

At the end of the treatments, a sufficient amount of blood was drawn while the animal was sedated (i.p. injection of xylazine, 8–12 mg/kg). The median canthus of each animal was punctured at a 45° angle using a capillary tube, and the blood samples were separated into two portions. One portion was collected in tubes containing 0.5 mg/mL EDTA dipotassium salt as an anticoagulant, which was carefully mixed to prevent RBC hemolysis for the assessment of hematological parameters. The other portion was left to coagulate at room temperature and refrigerated to remove clots. The clear serum was separated and centrifuged for 10 min at 3000 rpm and then transferred to an Eppendorf tube and kept at −20 °C until biochemical testing started. The ethics committee at Mansoura University recommended that rats should be euthanized by means of cervical dislocation. The kidney and liver were excised and washed, and two portions were collected as follows: liver tissue (0.5 g) was extracted, ground, and homogenized. Frozen 4.5 mL PBS (phosphate buffer saline (PBS; PH 7.5) was added, mixed well, and placed in Falcon tubes before being centrifuged at 825× *g* for 15 min at 4 °C. After careful separation and maintenance at −20 °C, the supernatant was used to determine the oxidative and hepatic antioxidant indicators [71]. As soon as was feasible, another liver tissue sample was fixed in 10% formalin for histological and immunohistochemical analysis.

### 4.5. Evaluation of Hematological Parameters

Whole blood samples were used to measure the number of leukocytes (TLCs) and erythrocytes (RBCs) and estimate the packed cell volume (PCV) and hemoglobin (Hb) content. The mean corpuscular volume (MCV), mean corpuscular hemoglobin (MCH), and mean corpuscular hemoglobin concentration (MCHC) were calculated in accordance with Feldman [72].

### 4.6. Evaluation of Serum Biochemical Parameters

The serum activities of alanine aminotransferase (ALT, catalog No.; AL 1031), aspartate aminotransferase (AST, catalog No.; AS 1061) (Biodiagnostics Company, Cairo, Egypt), and alkaline phosphatase (ALP, Ref: MD41233) (Spinreact Co., Santa Coloma, Spain), as well as levels of total, direct, and indirect bilirubin (Diamond, Cairo, Egypt) were evaluated. Total protein (catalog No.; SB-0250-500) and albumin (catalog No.; SB-028-500) were assessed using assay kits provided by the Stanbio Labs Company (TX, USA). To calculate A/G, albumin was divided by the concentration of globulin, which was obtained by subtracting albumin from total protein [73]. Serum creatinine (catalog No.; CR 1251) and serum urea (catalog No.; UR 2110) were estimated using kits supplied by the Biodiagnostics Company (Cairo, Egypt), while uric acid (catalog No.; MD41001) was measured following the protocol of the Spinreact Co. (Santa Coloma, Spain). All these tests were assayed spectrophotometrically (spectrophotometer, BM, Germany, 5010).

### 4.7. Assessment of Hepatic Lipid Peroxidation Indicators and Antioxidants in Hepatic Homogenates

Hepatic malondialdehyde (MDA, catalog No.; MD 25 29) [74] and antioxidant parameters superoxide dismutase (SOD, catalog No.; SD 25 21) [75], catalase (CAT, catalog No.; CA 25 17) [76], and glutathione-S- transferase (GST) [77], as well as reduced glutathione (GSH) [78], were assayed spectrophotometrically using commercially available Bio-Diagnostic ready-made kits (Cairo, Egypt) following the manufacturer’s protocols.

### 4.8. Cadmium Accumulation in Liver

Atomic absorption spectrophotometry (Perkin-Elmer A.A. Model 800) was used to assess the amount of Cd in the liver while accounting for background effects. For homogenization, digestion, and thorough drying, each sample (0.5 g) was treated with a 6:1 ratio of ultrapure concentrated nitric acid/perchloric acid on a hotplate. Before analysis and quantification using atomic absorption spectrophotometry, the samples were diluted with deionized water [79].

### 4.9. Evaluation of Liver Histopathology

To assess liver and kidney damage, histology tests were performed. Hepatic and renal tissues were preserved in 10% formaldehyde and then implanted in paraffin. The embedded tissue samples were sectioned (5 μm) and stained with hematoxylin and eosin in order to evaluate the general histological features. The tissue sections were viewed under a light microscope (Eclipse E200-LED; Nikon, Kawasaki, Japan) at a magnification of ×400 [80].

### 4.10. Expression of TNF-α and Caspase-3 via Immunohistochemistry

The sections were rehydrated in two containers and 100% ethanol for ten minutes each after the paraffin wax was submerged in three containers of fresh xylene for five minutes each. Subsequently, the sections were washed twice with distilled water. The process of creating crosslinks via formalin fixation involved soaking slides in sodium citrate buffer (PH 6.0), heating them to boiling point in a microwave, and then allowing them to cool on a bench for half an hour before staining with antibodies. Sections should be immersed in 3% H_2_O_2_ to reduce endogamous peroxidase activity, which could otherwise result in strong background staining, incorporating monoclonal rabbit anti-mouse primary antibody (dilution 1 in 100) and recombinant human TNF-α (ready to use), matching to their primary antibodies, incubated overnight at 4 °C in a humidified chamber, and then washed with Tris-buffered saline (TBS) for TNF-α and caspase-3, respectively. The process of “visualizing” the marking involved allowing the slides to sit at room temperature for five minutes while using 3,3′-diaminobenzidine tetrahydrochloride (DAB) from Dako, Glostrup, Denmark, as the chromogen. The slides were incubated with peroxidase-conjugated secondary anti-rabbit antibodies for half an hour at room temperature. Hematoxylin was used as a counterstain for the semi-quantitative analysis of the immunopositive response distribution pattern of the hepatic lobule using light microscopy (Olympus CX31, Shinjuku-ku, Tokyo, Japan). They were enumerated, and ImageJ analysis software (NIH, 1.46a, Bethesda, MD, USA) was used to assess their frequency per 1000 cells [81].

### 4.11. Statistical Analysis

The statistical analysis in this study was designed to ensure the validity, reliability, and interpretability of the results. The data were analyzed and presented as the means with standard errors (using Statistical Package for the Social Sciences (SPSS, version 17). All data were tested for normality via Levene’s test. The histogram figures were created using Excel (Microsoft Office 365, version 2020). A one-way analysis of variance (ANOVA) was used to evaluate the variance across groups. A *p*-value of less than 0.05 was regarded to indicate an acceptable level of significance.

## 5. Conclusions

The current study indicated that oxidative stress and apoptotic response play an essential role in the regulation of Cd-induced hepatic and renal injury. CLO co-treatment with Cd relatively mitigates the hepatic and renal toxicity of Cd, as proved by improvements in hematological and biochemical parameters and the attenuation of hepatic oxidative damage, inflammatory response, and hepatic apoptosis. The protective effect of CLO is mediated through its antioxidant, anti-inflammatory, and antiapoptotic activities. Further studies are needed to clarify the potent effect of clove oil against coronaviruses and cancer.

## Figures and Tables

**Table 1:** Phytochemical analysis of the clove oil sample.

**Oil sample**	**Phytochemical Analysis**	
	Phenolic Contents “mg gallic acid equivalent/g. oil”	Flavonoid Contents “mg catechin equivalent/g. oil”
	172.38	54.358

**Table 3:** GC–MS analysis of CLO.

Compound Name	Compound Class	Structure	Effects	Retention Time	Focus
**Eugenol**	Phenylpropanoid	C_10_H_12_O_2_	Anti-inflammatory, antioxidant, and antimicrobial properties	**16 min**	**76.8%**
**Beta-caryophyllene**	Sesquiterpene (Terpenes)	C_15_H_24_	Anti-inflammatory, analgesic, antioxidant, neuroprotective, anticancer, and antimicrobial properties	18 min	17.4%
**Alpha-caryophyllene**	Sesquiterpene (Terpenes)	C_15_H_24_	Anti-inflammatory, analgesic, and anticancer properties	19 min	2.1%
**Eugenol acetate**	Phenylpropanoid	C_10_H_12_O_2_	Anti-inflammatory, antioxidant, analgesic, and antimicrobial properties	21 min	1.2%

**Table 4:** Hematological results at the fourth week after clove oil treatment in rats intoxicated with cadmium (mean ± SE).

Group	RBCs (×10^6^/µL)	Hb (g/dL)	PCV (%)	MCV (fl)	MCH (pg)	MCHC (%)	WBCs (×10^3^/µL)
**Control**	6.74 ± 0.25 ^a^	14.76 ± 0.23 ^a^	40.60 ± 0.65 ^a^	59.46 ± 2.29 ^a^	21.96 ± 0.71 ^a^	36.97 ± 0.35 ^a^	17.33 ± 0.30 ^c^
**Cd**	5.42 ± 0.07 ^c^	13.02 ± 0.21 ^c^	34.86 ± 0.41 ^c^	59.60 ± 1.00 ^b^	22.26 ± 0.54 ^b^	37.34 ± 0.36 ^c^	20.29 ± 0.08 ^a^
**CLO**	6.86 ± 0.24 ^a^	14.57 ± 0.14 ^a^	40.92 ± 0.76 ^a^	59.26 ± 2.00 ^a^	20.56 ± 0.64 ^a^	34.74 ± 0.74 ^a^	17.15 ± 0.34 ^c^
**Cd+CLO**	6.08 ± 0.06 ^b^	13.59 ± 0.16 ^b^	39.37 ± 1.08 ^b^	60.56 ± 1.11 ^b^	21.83 ± 0.33 ^b^	36.06 ± 0.47 ^b^	19.57 ± 0.04 ^b^

Cd (cadmium), CLO (clove oil). Values with different superscript letters indicate significance at *p* < 0.05.

**Table 5:** Renal function outcomes in rats intoxicated with cadmium at the end of the fourth week following clove oil treatment (mean ± SE).

Group	Urea (mg/dL)	Uric Acid (mg/dL)	Creatinine (mg/dL)
**Control**	40.27 ± 0.64 ^c^	0.80 ± 0.02 ^c^	0.44 ± 0.006 ^c^
**Cd**	62.17 ± 0.61 ^a^	1.18 ± 0.04 ^a^	0.84 ± 0.011 ^a^
**CLO**	40.22 ± 0.64 ^c^	0.81 ± 0.015 ^c^	0.43 ± 0.0.007 ^c^
**Cd+CLO**	45.17 ± 0.30 ^b^	0.92 ± 0.011 ^b^	0.49 ± 0.014 ^b^

Cd (cadmium), CLO (clove oil). Values with different superscript letters indicate significance at *p* < 0.05.

**Table 6:** The hepatic homogenate of experimental rats treated with clove oil at the end of the fourth week showed indicators of oxidative stress and antioxidant biomarkers in rats intoxicated with cadmium (mean ± SE).

Group	MDA (nmol/g.Tissue)	SOD (U/g.Tissue)	CAT (U/g.Tissue)	GSH (mg/g.Tissue)	GST (U/g.Tissue)
**Control**	24.54 ± 2.87 ^c^	611.60 ± 0.92 ^a^	19.3 ± 0.07 ^a^	19.54 ± 1.42 ^a^	24.49 ± 0.59 ^b^
**Cd**	72.93 ± 6.19 ^a^	320.24 ± 44.09 ^c^	10.7 ± 0.16 ^c^	9.68 ± 0.55 ^c^	18.67 ± 0.56 ^d^
**CLO**	17.97 ± 2.92 ^c^	607.60 ± 2.24 ^a^	19.7 ± 0.007 ^a^	19.02 ± 1.56 ^a^	26.24 ± 0.54 ^a^
**Cd+CLO**	37.62 ± 3.62 ^b^	513.60 ± 12.34 ^b^	17.6 ± 0.03 ^b^	14.56 ± 0.68 ^b^	20.38 ± 0.26 ^c^

Cd (cadmium chloride), CLO (clove oil). Values with different superscript letters indicate significance at *p* < 0.05. MDA (malondialdehyde), SOD (superoxide dismutase), CAT (catalase), GSH (reduced glutathione), and GST (glutathione-S-transferase).

**Table 7:** Rats that were intoxicated with Cd and treated with CLO for four weeks showed changes in their liver’s Cd levels (mean ± SE).

Group	Cadmium Residues in Liver (mg/kg Liver)
**Control**	0.00013 ± 0.000042 ^c^
**Cd**	0.024496 ± 0.0036 ^a^
**CLO**	0.000346 ± 0.0001 ^c^
**Cd+CLO**	0.0333 ± 0.0051 ^b^

Cd (cadmium chloride), CLO (clove oil). Values with different superscript letters indicate significance at *p* < 0.05.

**Table 2:** Antioxidant activity of the clove oil sample as determined via the DPPH experiment using standard ascorbic acid.

	Sample Conc. (mg/mL)	% Remaining DPPH	IC50 (mg/mL)
**Oil sample**	0.168	49.72	0.1572
	0.084	59.94	
	0.042	75.71	
	0.021	85.23	
**Ascorbic acid**	0.062	15.267	0.0222
	0.031	39.084	
	0.016	61.069	
	0.008	74.809	

## Data Availability

All data are contained within the article.

## Abbreviations

CLO, clove oil; Cd, cadmium; ALT, alanine transaminase; AST, aspartate transaminase; ALP, alkaline phosphatase; SOD, superoxide dismutase; CAT, catalase; GSH, reduced glutathione; GST, glutathione-S- transferase; MDA, malondialdehyde; ROS, reactive oxygen species; TNF-α, tumor necrosis factor-alpha.
